# Tibial plateau fracture related to unicompartmental knee arthroplasty: Two case reports and literature review

**DOI:** 10.1097/MD.0000000000017338

**Published:** 2019-10-18

**Authors:** Chao Lu, Guozhu Ye, Wengang Liu, Huai Wu, Gaoyi Wu, Jin Chen

**Affiliations:** a Orthopedics Department, Guangdong Second Traditional Chinese Medicine Hospital; b Guangzhou University of Chinese Medicine, Guangzhou, Guangdong, China.

**Keywords:** plate support internal fixation, revision total knee arthroplasty, tibial plateau fracture, unicompartmental knee arthroplasty

## Abstract

**Rationale:**

Unicompartmental knee arthroplasty (UKA) is an effective method to treat single compartment disease of the knee joint. Report about the complications of UKA, especially tibial plateau fractures, is rare. Given its rarity, its pathogenesis is not well described, and a standard of treatment is still not established. Therefore, relevant studies and analysis of this complication have a significant effect on helping physicians avoid risks and guide clinical diagnosis and treatment.

**Patient concerns:**

The 1st case corresponds to a 70-year-old male patient who complained of knee pain, difficulty walking, nocturnal rest pain, and elevated skin temperature at 3 weeks after the left knee arthroplasty. The second case is a 72-year-old female patient who complained of left knee pain and swelling during movement at 2 weeks after the left knee arthroplasty.

**Diagnosis:**

The 1st case showed a fracture of the medial malleolus of the left knee and a secondary depression of the medial tibial plateau in X-rays and the second case showed a fracture of the medial malleolus of the left knee in computed tomography (CT) and X-rays.

**Interventions:**

The 1st case was treated with plate and screw fixation and the second case was treated conservatively and immobilized using brace and remained nonweight bearing for 6 weeks.

**Outcomes:**

After 1 year, both patients have good joint activity, and there was no pain or loosening of the prosthesis and fragment displacement.

**Lessons:**

The incidence of tibial plateau fractures (TPF) related to UKA might be low, but fatal and difficult to treat. Its pathogenesis determines procedure-related factors; when fracture develops, treatment should be based on the degree of displacement, stability of implant fixation, etc.

## 1 Introduction

The unicompartmental knee arthroplasty (UKA) is an effective method to treat single compartment disease of the knee joint. Its advantages include the following: small operation port, less bone loss, satisfactory postoperative activity, and quick postoperative recovery.^[1]^ With the improvement in prosthesis, surgical and technique, and further clarification of surgical indications, the efficacy rate is 93.2% in 10 years.^[2]^ With the increasing popularity of UKA surgery, its complications are increasing as well. One of its more serious complications is periprosthetic fracture around the tibial plateau.

The incidence of tibial plateau fractures (TPF) related to UKA is low. Currently, only a few detailed reports on this condition are available. Since the first case report was reported in 1997, there have been a total of 9 articles^[3–11]^ reported in literature (23 patients in total). Most were individual cases; hence, there were no statistical conclusions based on large sample to elucidate its pathogenesis and difficultly and guide its clinical treatment. Herein, this study will report on these 23 and 2 cases we experienced at our center. Disease characteristics, mechanisms, and treatment plans will be gathered and analyzed in detail to provide reference for clinical operations.

## 2 Materials and methods

We collected 2 cases of TPF after UKA treatment of osteoarthritis in our hospital who gave their written informed consent and related clinical reports with detailed clinical diagnosis and treatment data from 1997 to 2018. PubMed and Science Direct databases were searched using the following key words: unicompartmental knee replacement, UKA, tibial plateau fracture, and unicompartmental knee arthroplasty. Because of the low incidence, large sample summative studies and related randomized controlled trials are still lacking, and they all exist as case reports. Therefore, the included literature is mainly based on detailed records of the patient's condition, pathogenesis, treatment process, and follow-up articles. Then, patient's basic demographics, basic disease characteristics, mechanism of occurrence, and treatment plans were classified and statistically analyzed.

## 3 Case report

### 3.1 Case 1

**Figure 1:**
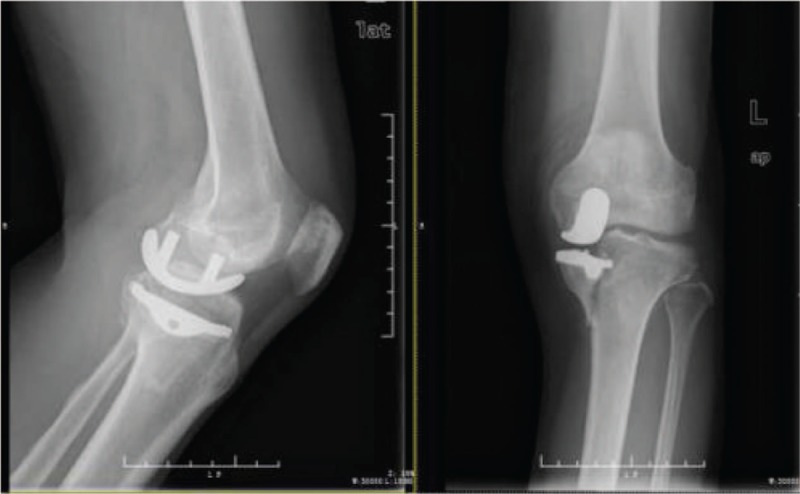
Preoperative radiograph showing medial malleolus fracture of the left knee, followed by a slight downward shift of the medial tibial plateau.

**Figure 2:**
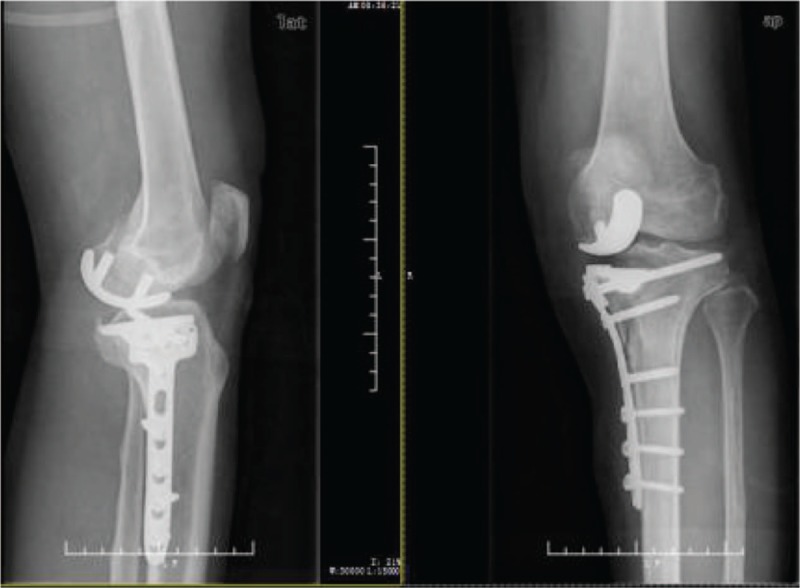
Review of X-rays on the first postoperative day. Plates and screws were well applied. The screws were in good position and did not interfere with the position of the prosthesis.

**Figure 3:**
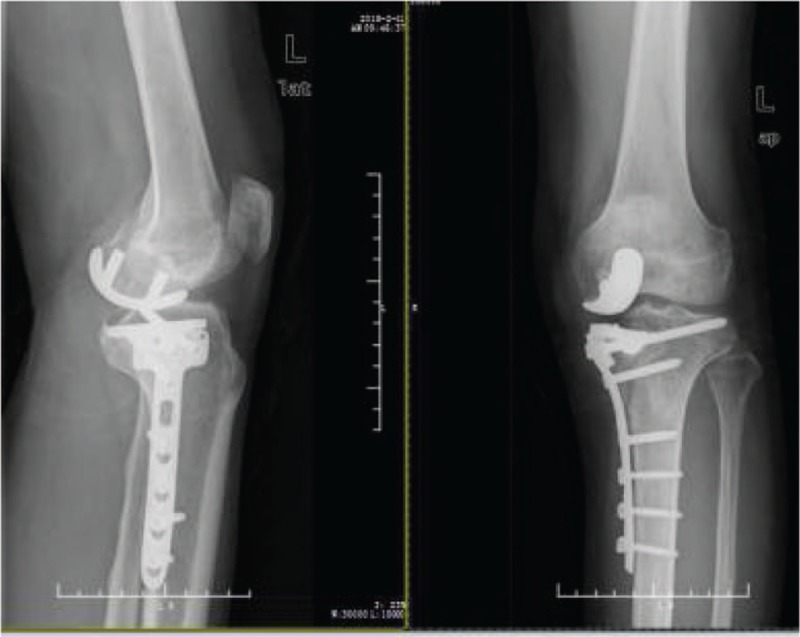
X-rays after 1 year.

A 70-year-old male patient had left knee pain for more than 10 months. X-rays showed medial compartment knee arthritis and osteoporosis. Left knee arthroplasty was performed. The patient was assisted with a walker on the first postoperative day. He was discharged 1 week later and was free to walk when discharged. His knee range of motion was brought back to normal. At 3 weeks after the operation, he complained of knee pain, difficulty walking, nocturnal rest pain, and elevated skin temperature, but not obvious causes were noted. The patient had no exudation, swelling, activity disorder, or obvious deformity. X-rays showed a fracture of the medial malleolus of the left knee and a secondary depression of the medial tibial plateau (Fig. 1). The diagnosis was UKA-related TPF. Internal fixation of the medial plate was performed (Fig. 2). During the surgery, fracture of the medial plateau of the left tibia, large palpebral fissure, and fracture line were approximately 4 cm below the platform. About 4-cm local cortical fracture was found displaced below the platform, the platform was submerged approximately 1 mm, and the platform gasket was not loosened. The medial anatomic plate of the tibial plateau (6 volumes) was used, and 8 screws were used to fix the plate. Four proximal locking screws (only 1 spike could be placed to avoid the keel and provide support) were used. Braking was performed for 6 weeks after surgery. After 1 year (Fig. 3), the joints had good range of motion, there were no loosening of the prosthesis and fragment displacement, and the HSS (Hospital for Special Surgery) score was 15.

### 3.2 Case 2

**Figure 4:**
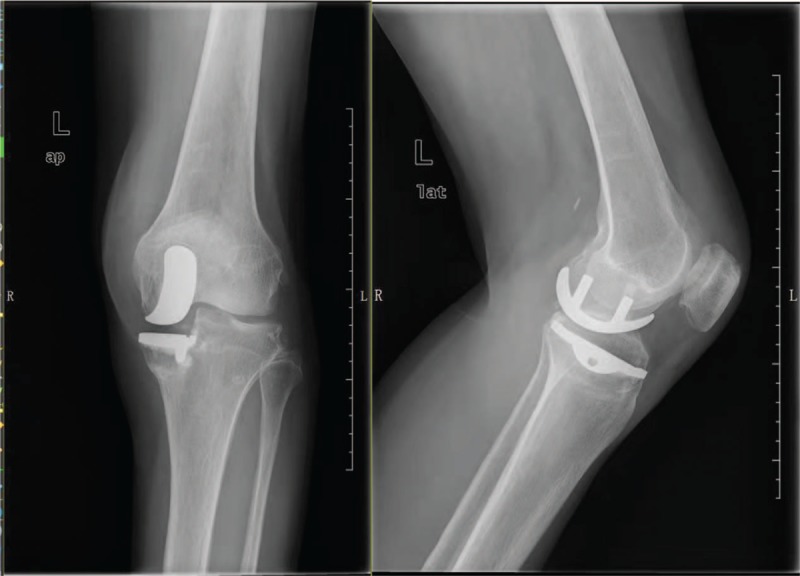
X-rays showing medial malleolus fracture of the left knee.

**Figure 5:**
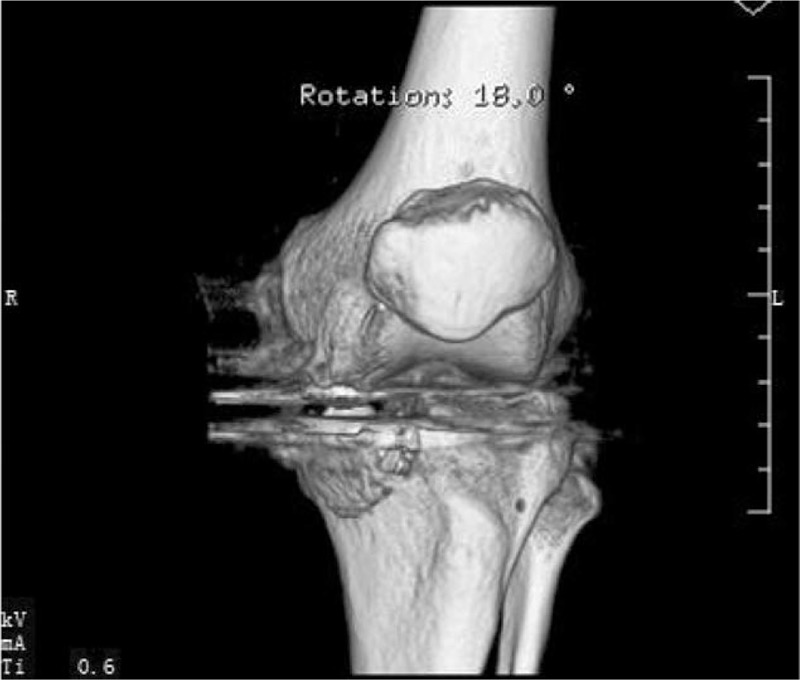
CT showing medial malleolus fracture of the left knee. CT = computed tomography.

**Figure 6:**
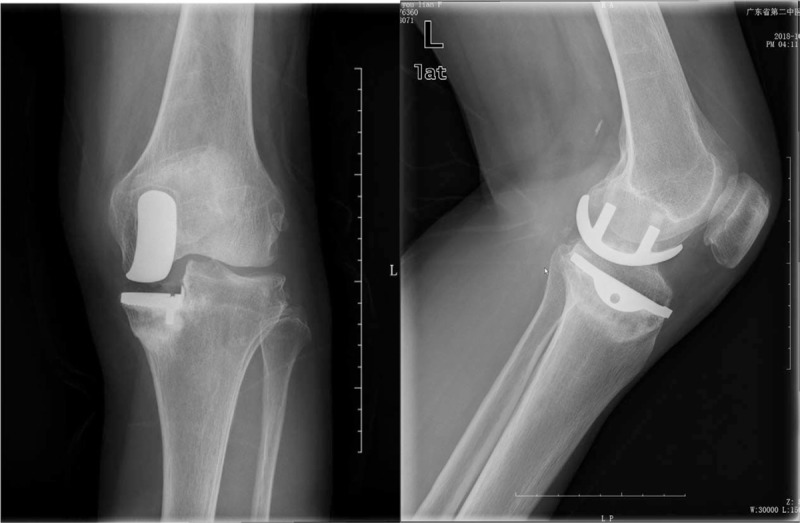
Radiograph after 1 year showing the fracture lines are better.

A 72-year-old female patient had bilateral knee pain for more than 2 months. X-rays showed medial compartment knee arthritis and osteoporosis. UKA was performed for both knees. There were no postoperative discomforts. After 2 weeks, left knee pain and swelling were felt during movement. The patient had no exudation, activity disorder, or obvious deformity. Computed tomography (CT) and X-rays showed a fracture of the medial malleolus of the left knee (Figs. 4 and 5). The diagnosis was UKA-related TPF. She was treated conservatively and immobilized using brace and remained nonweight bearing for 6 weeks. After 1 year (Fig. 6), X-rays showed good joint activity, and there was no pain or loosening of the prosthesis. At the same time, pain relief was achieved despite full weight-bearing while standing. At present, this case is still being followed.

## 4 Discussion

UKA is currently the latest technology in the field of knee joints. For patients with single compartment disease, its surgical effect is highly satisfactory. With the continuous popularization of surgical techniques, research on the prevention and treatment of complications is urgent and significant. TPF is a serious complication of UKA. There is no unified conclusion about its pathogenesis and treatment plan. The two presented cases indicate that choosing the right treatment can achieve a good prognosis. This study analyzes the characteristics, mechanisms, and treatment options of tibial plateau periprosthetic fractures related to UKA to provide guidance for clinical practice.

### 4.1 Incidence characteristics

Table 1 shows the characteristics of tibial plateau periprosthetic fractures related to unicompartmental knee arthroplasty.

**Table 1:**
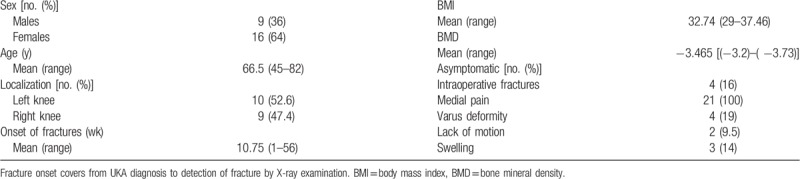
The characteristics of tibial plateau periprosthetic fractures related to unicompartmental knee arthroplasty.

### 4.2 Pathogenesis

In all 25 cases, the causes were numerous. Four cases of intraoperative fractures were mainly caused by the excessive hammering force. The main manifestation was the visible fracture line during the operation. Fractures originated from the sagittal osteotomy or keel groove base. Among the 21 patients with postoperative fractures, the cause of injury was not explained in 1 patient. Others were considered to have been caused by fatigue, physical therapy, obesity, and osteoporosis. The pathogenesis includes bone marrow necrosis resulting in insufficient supporting force, insufficient keel groove preparation, fixed nail injury on the medial cortex of the tibia, residual varus deformity, and inappropriate size or position of the tibial component. Table 2 shows the causes and mechanisms of tibial plateau periprosthetic fractures related to UKA.

**Table 2:**
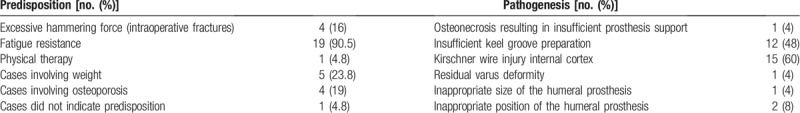
Causes and mechanisms of tibial plateau periprosthetic fractures related to unicompartmental knee arthroplasty.

#### 4.2.1 Local bone necrosis leads to weakened platform support

In 1997, Kumar and Fiddian^[3]^ first reported a case of fracture of the medial tibial plateau after UKA. During the revision procedure, most osteolyses were found in the medial plateau. Necrosis was due to insufficient prosthesis support and excessive residual cortical pressure. This causes stress fractures, and the cause of osteonecrosis is unclear.

#### 4.2.2 Insufficient tibial preparation, improper sagittal osteotomy, or keel preparation

Some scholars believe that the TPF during UKA and early postoperative period have an important relationship with surgical techniques. Sloper et al,^[4]^ Van Loon et al,^[7]^ and Pandit et al^[10]^ reported a total of 4 cases of intraoperative TPF that occurred during implantation of the tibial plateau components. During the assembly, the surgeon hammered the tibial plateau prosthesis resulting in split fracture. This was due to the inadequate preparation of the tibia. There are 2 interrelated mechanisms. First, it was too deep to make a keel groove. The posterior keel groove extends to the posterior cortex of the humerus, causing a small notch in the posterior cortex of the tibial plateau to weaken the bearing capacity of the posterior cortex. In this mechanism, the fracture line often starts from the posterior keel groove.^[4]^ Second, the sagittal osteotomy is employed when the reciprocating saw is used. Excessive damage to the posterior cortex leads to reduced local support, stress concentration, and insufferable violent hammering. In this mechanism, the fracture line often starts from the posterior of sagittal osteotomy.^[8]^ Some scholars have also confirmed the mechanism of this situation through biomechanical experiments. Clarius et al^[12]^ believed that the prolonged sagittal osteotomy will weaken the posterior cortical bone and cause the tibial plateau to withstand the maximum load, which may lead to fractures of the tibial plateau. It is also speculated that the lower the bone mineral density (BMD), the greater the fracture risk. The maximum load during fracture is closely related to BMD.

#### 4.2.3 Inappropriate size or position of the tibial component

Van Loon et al^[7]^ believe that the size of the tibial osteotomy and the appropriate size of the tibial plateau is very important. An oversized platform will cause some of the force to be applied to the nonsupported part of the tibial tray, resulting in a stress fracture. The platform being too small or placed in an aside position will result in uneven load transfer between the tibial component and the tibial. Stress can be concentrated in a small, eccentric area of the tibia, resulting in a stress fracture of the tibial plateau. The sagittal position of the tibial plateau prosthesis is also very important. Different backtilt angles will cause abnormal force distribution in the anterior and posterior areas of the platform. If the support strength is not uniform under the combined platform, prosthesis loosening or platform fracture is likely to occur. Seon et al^[9]^ speculate that the position of the sagittal prosthesis is very important. The prosthesis is placed behind and is not accurately placed on the front cortical bone, so the frontal platform support is relatively weak. In addition, when the platform is tilted and too reclined, the stress distribution on the front side will cause the front of the platform to sink, and various factors will cause pressure fracture inside the tibial plateau.

#### 4.2.4 Weakening of nail hole in the proximal tibia

In UKA surgery, tibial preparation often needs standard guide of bone cutting auxiliary, so that screw fixation will leave a hole diameter of more than 3 mm, breaking the integrity and weakening the compressive capacity of the cortex, which is one of the causes of fracture. At the same time, many reports support this theory. Lindstrand et al^[13]^ introduce the fracture of the tibial plateau after UKA in a multicenter study. They emphasized that the farther the distal tibia hole to the medial cortex, the more serious its affect will be to cause compressive ability. Yang et al^[5]^ reported 2 cases: the proximal tibia fixed nail is too close to the medial cortex, weakening the cortex, increasing local stress, and occurrence of postoperative fracture. In this case, we can observe that fracture line often passes through a fixed orifice, especially in case of delayed healing of nailing, and stress distribution will persist. Brumby et al ^[14]^ insist that use of numerous guide plates to fix screw holes in single condyle replacement may be the main cause of stress fracture in tibial plateau. They believe that it is very dangerous to use more than 3 nail holes as one of them may damage the medial cortex. These fractures usually occur between 3 and 18 weeks after the surgery (mean, 8 weeks). Especially, for novice doctors, repeated positioning of the osteotomy leads to excessive residual hole in the proximal tibia, which will significantly reduce the maximum stress load of the tibial plateau and thus the stress fracture. This theory is also confirmed in some basic experiments.

#### 4.2.5 Residual varus deformity

The medial compartment assumes a total load of 54.6% in normal knee medial compartment model, and some studies showed a total of 59.7% in UKA medial compartment model and 40.3% for lateral compartment, which illustrates that the stress distribution in the medial compartment is higher than normal. As the angle of the knee joint increases, the stress distribution in the region is also increasing. Seon et al^[9]^ declare that the ability of UKA to correct varus deformity is inferior to that of total knee arthroplasty (TKA), which is limited to 5°. In addition, improper selection of patients who has severe varus causes residual varus stress and excessive medial stress. The postoperative load was focused on the medial side, and when the tibia was prepared, the subchondral bone of the tibia could further lead to insufficient support in the platform, which resulted in a stress fracture. Therefore, it is often recommended to choose high tibial osteomy or TKA treatment for patients with obvious introversion.

#### 4.2.6 Inappropriate case selection and proximal tibia subchondral bone support

It is very important to grasp strictly the surgical indications for UKA: the medial compartment degeneration of the knee is a suitable case for UKA. In terms of the degree of degeneration, “bone-on-bone” change is the best surgical indication, and long-term “bone-on-bone” wear will cause medial compartment sclerosis of the subchondral bone. Compared with the cancellous bone, the support force of the platform prosthesis was increased, and the stability of the prosthesis was higher; therefore the incidence of fracture was decreased. Therefore, it is very important to grasp the strict indications in the selection of surgical cases, because increasing indications means more complications.

#### 4.2.7 Excessive tibial osteotomy and insufficient support in the platform

When the tibia is prepared, excessive osteotomy will lead to the reduction of bone mass in the platform and insufficient support in the platform, and maximum load reduction on the medial platform will increase the risk of fracture. During the operation, the bone should be kept as far as possible.

### 4.3 Treatment plan for UKA-related TPF

Given all the case data, the treatment plan, including conservative treatment, support plate internal fixation, knee joint revision, tension screw, among others are explained (Table 3).

**Table 3:**
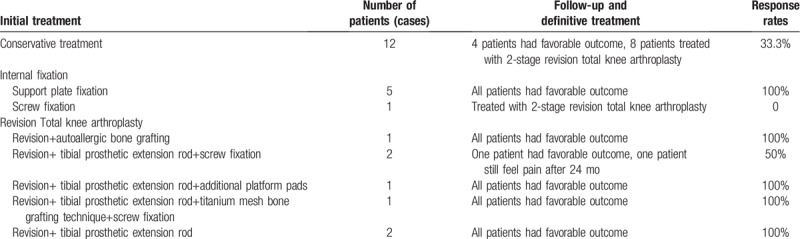
Treatment plan and effect of fracture treatment of tibial plateau periprosthetic fractures related to unicompartmental knee arthroplasty.

#### 4.3.1 Conservative treatment

Patients with UKA intraoperative or postoperative plateau fractures have high failure rates with conservative treatment. Clinically, conservative treatment should be based on fracture displacement and stability of the prosthesis. If there is no apparent displacement of the fracture, the joints are not significantly internal. If there was no evidence of loosening of the platform prosthesis by turning over the deformity, conservative treatment can be chosen. The conservative treatment plan is mainly to repair and fix the brace with TKA, avoid weight-bearing for 6 weeks, and exercise the appropriate joint by flexion and extension exercises. Because of the long period of conservative treatment, due to the lack of a strong fixation method, it is prone to fracture repositioning, fracture nonunion, or even loosening of the prosthesis. The failure rate is high. Once failure is found in the follow-up visit, prompt repair of surgery is required. Of the 25 patients in this group, 4 patients who achieved good prognosis through conservative treatment had strict screening criteria and strict treatment options (Table 4).

**Table 4:**
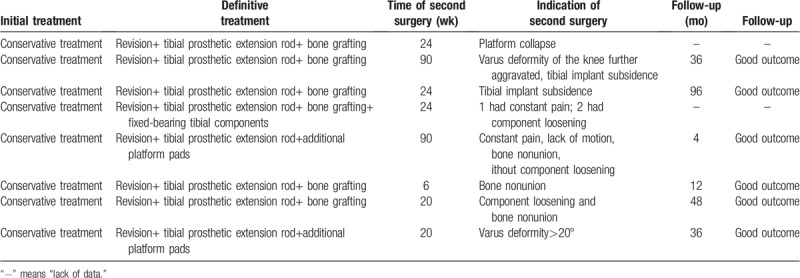
Characteristics of failures in conservative treatment of tibial plateau periprosthetic fractures related to unicompartmental knee arthroplasty.

#### 4.3.2 Support plate fixation

Plate and screw fixation is an important fixation method for the fracture of the platform. The treatment can be selected when the prosthesis is stable, and the fracture can be dissected and reset. The medial support plate of the tibial plateau is preferred. The technical difficulty lies in the fracture of the UKA platform. Because the keel is blocked, insertion of long screws is very difficult. Usually, only one long screw can be used to fix the rest. Only the short screws can be used for locking and supporting. When drilling, it is necessary to prevent loosening of the prosthesis. A total of 5 patients in this group were treated with plate and screw internal fixation. The follow-up was satisfactory, and there was no failure of internal fixation.

#### 4.3.3 Knee revision

Most scholars have opted for a knee revision treatment. This is a reliable method for patients with significant fracture displacements, prosthesis loosening is preferred, if the patient initially chooses conservative treatment but the symptom persists for more than 3 months and had not improved, if varus malformation is aggravated during follow-up, fracture displacement increases, the fracture does not heal, or the prosthesis sinks and loosens, etc. Surgery requires the choice of a tibial prosthetic extension rod to increase prosthesis stability and distribute platform stress. For the medial platform, the bone defect can be treated according to the defect area through structural bone grafting, screw cement filling techniques, and additional platform pads. For special conditions, the fracture around the comminuted humeral prosthesis can be resolved using titanium mesh + autograft/allograft bone grafting technique during TKA revision. For early detection of TPF during surgery, TKA revision combined with screw fixation can be used. Of the 25 patients in this group, 16 patients eventually achieved good healing through TKA revision therapy. This treatment is the most stable treatment.

## 5 Conclusion

TPF is a serious complication after UKA. With the widespread use of UKA, the incidence is believed to increase. However, complications are serious and difficult to treat. It will become a major challenge for arthrologists. Determining its prevention, early diagnosis, and treatment is an urgent problem. We intend to analyze and introduce the successful diagnosis and treatment experience of 2 patients in our hospital and combine domestic and foreign studies of 25 cases with detailed contents for analysis. Moreover, we summarized the characteristics of the fracture, pathogenesis, and the general rule of treatment. This provides an objective reference standard for its diagnosis and treatment for clinical reference. Strict control of UKA indications, selection of appropriate patients for surgery, careful operation during operation, and avoidance of operations that lead to excessive damage to the cortex around the platform can effectively avoid this complication. Once a TPF has occurred, the treatment needs to be selected based on the combination of fracture pattern, displacement, varus, and prosthesis stability. Conservative treatment has a high failure rate and requires careful selection, requiring a longer observation period after treatment. Internal fixation and TKA revision are reliable treatment options and follow-up results are good.

## Author contributions

**Data curation:** Wengang Liu, Huai Wu, Gaoyi Wu, Jin Chen.

**Supervision:** Wengang Liu.

**Writing – original draft:** Chao Lu, Guozhu Ye, Huai Wu, Gaoyi Wu, Jin Chen.

**Writing – review & editing:** Guozhu Ye, Jin Chen.
